# Sustained Maternal Hyperandrogenism During PCOS Pregnancy Reduced by Metformin in Non-obese Women Carrying a Male Fetus

**DOI:** 10.1210/clinem/dgaa605

**Published:** 2020-09-01

**Authors:** Frida Andræ, David Abbott, Solhild Stridsklev, Anne Vibeke Schmedes, Ingrid Hov Odsæter, Eszter Vanky, Øyvind Salvesen

**Affiliations:** 1 Department of Obstetrics and Gynecology, Nordlandssykehuset, Bodø, Norway; 2 Wisconsin National Primate Research Centre, University of Wisconsin, Madison, WI; 3 Department of Obstetrics and Gynecology, St Olavs hospital, Trondheim University Hospital, Trondheim, Norway; 4 Department of Clinical and Molecular Medicine, Norwegian University of Science and Technology, Trondheim, Norway; 5 Biochemistry and Immunology, Lillebælt Hospital, Vejle, Denmark; 6 Department of Clinical Chemistry, St Olavs Hospital, Trondheim University Hospital, Trondheim, Norway; 7 Faculty of Medicine and Health Sciences, Norwegian University of Science and Technology, Trondheim, Norway

**Keywords:** PCOS, pregnancy, metformin, androgens, testosterone, androstenedione, gender, obesity

## Abstract

**Context:**

Large, longitudinal studies on androgen levels in pregnant women with polycystic ovary syndrome (PCOS) are lacking. While metformin has a mild androgen-lowering effect in non-pregnant women with PCOS, its effects on maternal androgen levels in pregnancy are less well understood.

**Objective:**

To describe androgen patterns in pregnant women with PCOS and in healthy control women, and to explore the potential effects of metformin on maternal androgen levels in PCOS.

**Design and Setting:**

A post hoc analysis from a randomized, placebo-controlled, multicenter study carried out at 11 secondary care centers and a longitudinal single-center study on healthy pregnant women in Norway.

**Participants:**

A total of 262 women with PCOS and 119 controls.

**Intervention:**

The participants with PCOS were randomly assigned to metformin (2 g daily) or placebo, from first trimester to delivery.

**Main Outcome Measures:**

Androstenedione (A4), testosterone (T), sex-hormone binding globulin (SHBG), and free testosterone index (FTI) at 4 time points in pregnancy.

**Results:**

Women with PCOS versus healthy controls had higher A4, T, and FTI, and lower SHBG at all measured time points in pregnancy. In the overall cohort of women with PCOS, metformin had no effect on A4, T, SHBG, and FTI. In subgroup analyses, metformin reduced A4 (*P* = 0.019) in nonobese women. Metformin also reduced A4 (*P* = 0.036), T (*P* = 0.023), and SHBG (*P* = 0.010) levels through pregnancy in mothers with a male fetus.

**Conclusion:**

Metformin had no effect on maternal androgens in PCOS pregnancies. In subgroup analyses, a modest androgen-lowering effect was observed in nonobese women with PCOS. In PCOS women carrying a male fetus, metformin exhibited an androgen-lowering effect.

Polycystic ovary syndrome (PCOS) is a common endocrine and metabolic disorder. When using Rotterdam criteria, it effects up to 15% of women in reproductive age (1–3). It is also associated with overweight, obesity, and insulin resistance (4). Pregnant women with PCOS are hyperandrogenic and have more pregnancy complications, such as miscarriage, preterm birth, gestational diabetes, and pre-eclampsia (5–8). Interest in metformin treatment, an insulin-lowering drug, for women with PCOS increased when it became evident that insulin resistance plays an important role in the pathophysiology of the disorder (9). Early trials in women with PCOS demonstrated modest benefits in weight reduction, decreased serum androgens, and restoration of regular menstrual cycles in approximately 50% of women with oligomenorrhea (10, 11). There are, however, only a few published studies on androgen levels throughout PCOS pregnancies and the effects of metformin treatment during pregnancy, and none reach randomized controlled trial rigor (11, 12).

Placental aromatase (*CYP19A1*) is thought to protect the fetus against maternal androgens, from their metabolism to estrogens or bioinactive conjugates. Maternal hyperandrogenism in PCOS gestations may breach a compromised placenta and affect the developing offspring. Diminished placental expression of *CYP19A1* and *HSD3B1* during PCOS gestation likely impair placental metabolism of androgens (13). Perhaps not surprisingly, PCOS placentae exhibit structural and molecular dysfunction (14, 15), including increased signal transducers and activators of transcription 3 phosphorylation, indicating that specific metabolic pathways are activated by maternal gestational hyperandrogenism (16). There is evidence that maternal androgens may modulate the programming of placental and fetal steroidogenesis, and alter circulating androgen levels in utero (17, 18). Human studies have demonstrated traits of in utero androgenisation of female offspring (19–22). In mice, prenatal androgen exposure, alone, plays an important role in intergenerational and transgenerational susceptibility to PCOS (23).

Whether increased maternal androgens are useful as risk markers for short- and long-term consequences to human offspring health, remains to be fully explored.

The aims of our study were (1) to describe androgen patterns during pregnancy in women with PCOS and relate them to androgen levels during pregnancy in healthy control women, (2) to explore whether metformin alters androgen levels during pregnancy in women with PCOS, and (3) to ascertain whether PCOS maternal androgen levels differ according to body mass index (BMI) category and sex of the fetus.

## Material and Methods

### The PregMet study

In all, 274 women with PCOS participated in the original PregMet study (24). In 1 patient, a partial 21-hydroxylase deficiency had been overlooked, and she was excluded after randomization. Seventeen women participated with 2 pregnancies. In 11 women, serum samples were not available for analyses. In all, serum samples from 262 participants were analyzed.

**Figure 1.**
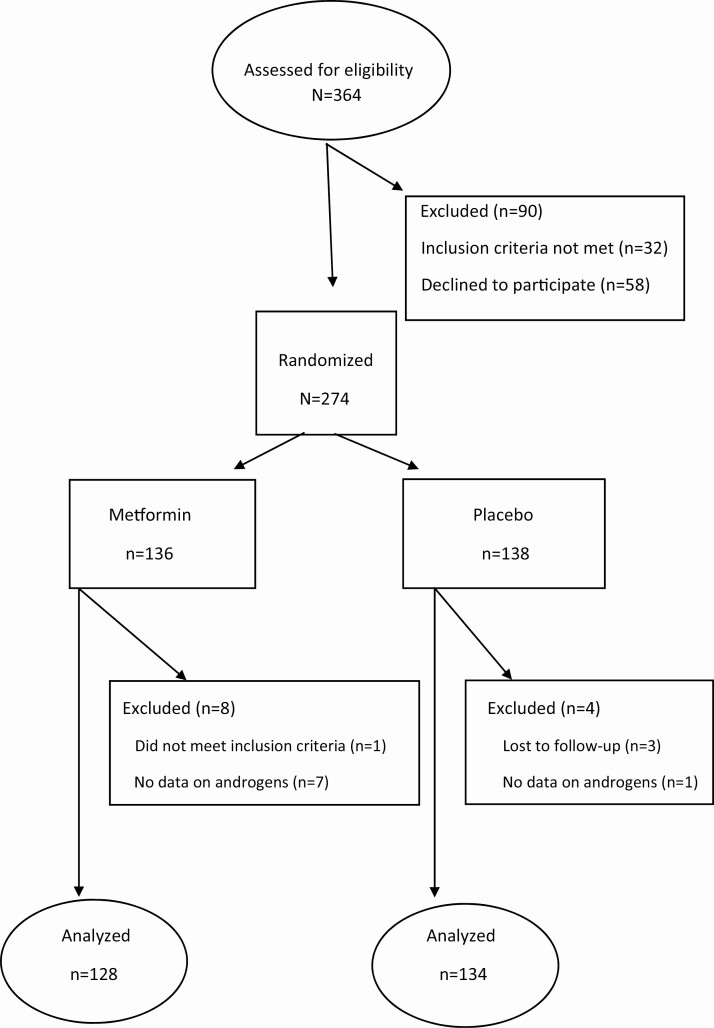
Flow chart on enrollment and randomization of pregnant women with polycystic ovary syndrome (PCOS).

Participants were recruited from 11 study centers in Norway. Inclusion criteria for the PregMet study were: (1) PCOS diagnosed according to the Rotterdam criteria (25), (2) age 18–45 years, (3) gestational age between 5–12 weeks, and (4) a single viable fetus. The exclusion criteria were alanine aminotransferase higher than 90 IU/l, serum creatinine concentration higher than 1.70 mg/dl, known alcohol abuse, previously diagnosed diabetes mellitus or fasting serum glucose > 126 mg/dl (6.9 mmol/l) at the time point of inclusion, treatment with oral glucocorticoids, or use of drugs known to interfere with metformin. The participants were randomized to either 2000 mg metformin daily or placebo (Fig. 1).

All participants received counseling on lifestyle and diet at inclusion. To counteract possible metformin effects on folate or vitamin B levels, the participants were advised to take 0.8 mg folate and 1 multivitamin tablet daily, throughout pregnancy. An intake of more than 85% of the prescribed tablets was self-reported by 80% of the participants and was considered as good/acceptable compliance. Randomization, stratification, and blinding for the study allocation is described elsewhere (24). Blood samples were collected at inclusion to the study and at gestational weeks 19, 32, and 36. The study was carried out between 2005 and 2009. Twelve women dropped out, that is, discontinued medication and did not turn up at scheduled visits. Participants and all investigators were blinded to group assignment.

### NormalFlow study

A total of 124 women were included at St. Olavs Hospital, Trondheim University Hospital between June 2008 and May 2010. The study recruited healthy women with an ongoing first trimester, single pregnancy, and aged 18–38 years old (26). Exclusion criteria were: (1) somatic or mental disease (eg, diabetes, kidney, cardiovascular diseases, and PCOS), (2) pregnancy complications in previous pregnancies (eg, pre-eclampsia, intrauterine fetal death, gestational diabetes mellitus, preterm delivery), and (3) multiple pregnancies. Missed abortions and congenital anomalies were excluded. In addition, 5 women were excluded from the original NormalFlow cohort before analyses due to previously undetected polycystic ovary syndrome (n = 1), pre-eclampsia (n = 3), and intrauterine fetal death in week 35 (n = 1), leaving 119 women for analysis. Blood samples were available from gestational weeks 11, 13, 19, and 24.

In both studies (24, 26), androgen levels were originally analyzed by the enzyme-linked immunosorbent assay (ELISA)-method and androstenedione (A4) and testosterone (T) were later re-analyzed by liquid chromatography mass spectrometry. Plasma samples were extracted by supported liquid extraction and the eluate was evaporated and reconstituted before analysis on liquid chromatography mass spectrometry. The analysis was calibrated by in-house prepared calibrators and the relative standard deviation was below 10%. Quality was assured by monthly participation with satisfactory results in the external quality control program for steroid hormones from NEQAS, United Kingdom. Sex-hormone binding globulin (SHBG) was analyzed by the ELISA technique, with the reagents and calibrators supplied by the manufacturer (DRG Instruments GmbH, Marburg/Lahn, Germany).

Free testosterone index (FTI) was calculated (T/SHBG) × 100.

### Statistical analysis

Maternal baseline characteristics at inclusion for the PregMet study were summarized using mean and standard deviation for continuous variables, and counts and proportions for categorical variables.

Androgen levels during pregnancy were examined using linear mixed models. The logarithm of the androgen measurements was taken as response variables, since these values were approximately normally distributed. The covariance matrix for the repeated measurements for each woman were taken as unstructured due to apparent heteroscedasticity.

The effects of metformin on androgen levels were tested for significance, comparing areas under curve for the different treatment groups.

Subgroup analyses were performed according to BMI category under and over 30 kg/m^2^, and according to the sex of the fetus.

The significance level was taken as 5%. All analyses were done using R version 2.13.1.

### Funding

The Liaison Committee between the Central Norway Regional Health Authority and the Norwegian University of Science and Technology funded the original study. Metformin and placebo tablets were delivered free of charge by Weifa A/S, Oslo. There was no specific funding for this substudy.

## Results

At baseline, women with PCOS were older, with a higher BMI and blood pressure compared with healthy controls (Table 1). There was no difference in baseline characteristics between women with PCOS randomized to metformin or placebo treatments (Table 1).

**Table 1.** Baseline characteristics of women with PCOS according to randomization to metformin or placebo and healthy controls in the first trimester of pregnancy

	All PCOS Participants (N = 262)	PCOS Randomized to Metformin (N = 128)	PCOS Randomized to Placebo (N = 134)	Healthy Controls (N = 119)	Metformin vs Placebo (*P*-value)	All PCOS vs Controls (*P*-value)
Age, years (SD)	29.5 (4.4)	29.7 (4.4)	29.2 (4.4)	27.9 (4.2)	0.343	0.001
Height, cm (SD)	167.5 (5.6)	167.2 (5.7)	167.8 (5.5)	168.2 (5.9)	0.410	0.292
Weight, kg (SD)	80.7 (19.0)	82.9 (20.5)	78.7 (17.3)	68.0 (13,0)	0.073	0.000
BMI, kg/m^2^ (SD)	28.9 (7.1)	29.6 (7.2)	28.3 (6.9)	24.0 (4.3)	0.115	0.000
SBP (mmHg)	118 (12)	119 (12)	117 (11)	114 (12)	0.176	0.002
DBP (mmHg)	73 (11)	74 (12)	73 (10)	68 (10)	0.272	0.000
Smoking (%)	21 (8.0)	12 (9.4)	9 (6.8)	12 (11.2)	0.439	0.334
Metformin at conception (%)	83 (31.7)	41 (32.0)	42 (31.3)	0	1.00	–
GDM at inclusion no (%)^*a*^	22 (8.4)	9 (7.0)	13 (9.7)	0	0.508	–
**Ethnicity (%)**	–	–	–	–	–	–
Caucasian	257 (98.1)	124 (96.9)	133 (99.3)	119 (100)	0.528	0.681
**Working status (%)**	–	–	–	–	0.377	0.000
Working	221 (80.4)	111 (87.4)	110 (82.1)	90 (80.4)	–	–
Student	10 (3.8)	5 (3.9)	5 (3.7)	16 (14.3)	–	–
Other	30 (11.5)	11 (8.6)	19 (14.2)	6 (5.4)	–	–
**Phenotype (%)**	–	–	–	–	0.908	–
HA+PCOM+OA	–	76 (59.4)	82 (61.2)	–	–	–
HA+PCOM	–	13 (10.2)	13 (9.7)	–	–	–
HA+OA	–	6 (4.7)	4 (3.0)	–	–	–
PCOM+OA	–	33 (25.8)	35 (26.1)	–	–	–

Abbreviations: BMI, bodymass index; DBP, diastolic blood pressure; GDM, gestational diabetes; HA, hyperandrogenism; OA, oligo-amenorrhea, PCOM, polycystic ovary morphology; PCOS, polycystic ovary syndrome; SBP, systolic blood pressure; SD, standard deviation.

^*a*^GDM = gestational diabetes mellitus diagnosed according to the 1999 World Health Organization criteria, as the PregMet study was performed in 2005–2009.

PCOS versus healthy controls

**Figure 2.**
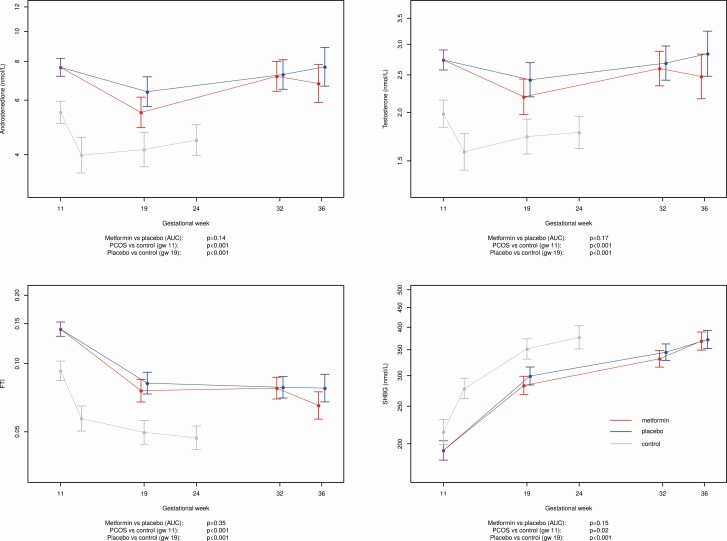
Androstenedione (**a**), testosterone (**b**), free testosterone index (FTI) (**c**), and sex-hormone binding globulin (SHBG) (**d**) in women with polycystic ovary syndrome (PCOS), treated with metformin versus placebo in pregnancy and healthy pregnant controls.

In both the first and second trimester of pregnancy, and compared with healthy control mothers, serum A4, T, and FTI were higher in mothers with PCOS regardless of placebo or metformin treatment. Sex-hormone binding globulin levels were lower at all time points in both metformin- and placebo-treated PCOS compared with healthy controls (Fig. 2A–2D).

Placebo-treated PCOS population

In all women with PCOS treated with placebo, A4 and T levels were relatively stable throughout pregnancy, that is, gestational weeks 11, 19, 32, and 36 (Fig. 2A–2B). Free testonsterone index decreased from early to mid- and late pregnancy (Fig. 2C), while SHBG levels increased during the first half of gestation (Fig. 2D).

PCOS cohort metformin versus placebo

For the total PCOS cohort, there were no differences in levels of A4 (*P* = 0.14), T (*P* = 0.17), SHBG (*P* = 0.35), and FTI (*P* = 0.15) in the metformin versus placebo PCOS groups throughout gestation.

Subgroup analyses according to BMI

At inclusion, nonobese PCOS women had higher levels of A4 (*P* = 0.001) and SHBG (*P* = 0.001), and lower levels of FTI (*P* = 0.047) compared with obese women with PCOS (BMI > 30 kg/m^2^), and no difference in T levels.

**Figure 3.**
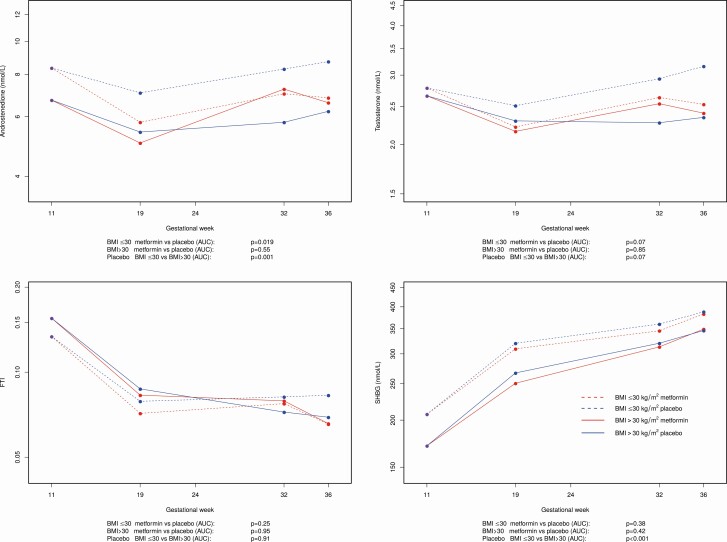
Androstenedione (**a**), testosterone (**b**), free testosteroneindex (FTI) (**c**), and sex-hormone binding globulin (SHBG) (**d**) in women with polycystic ovary syndrome (PCOS), treated with metformin versus placebo in pregnancy according to body mass index (BMI) category.

Metformin significantly lowered A4 (*P* = 0.019) and tended to lower T (*P* = 0.07) levels in nonobese women with PCOS, while it had no effect on obese PCOS women. Free testosterone index values were not significantly altered by metformin in either obese or nonobese women with PCOS (Fig. 3A–3D).

Subgroup analyses by sex of the fetus

**Figure 4.**
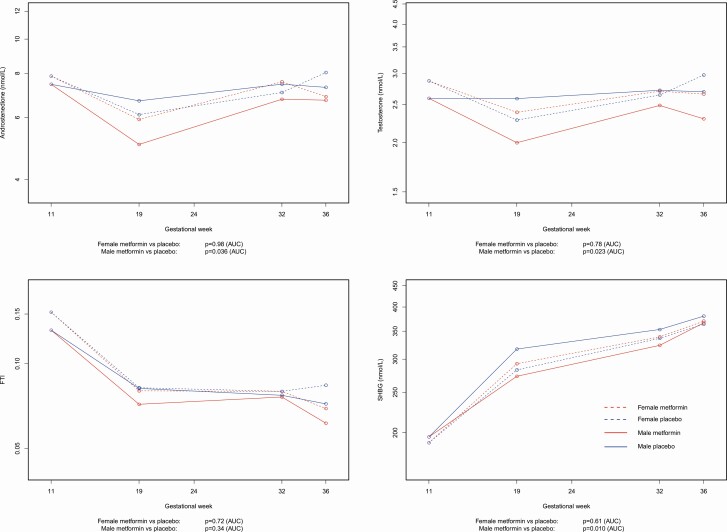
Androstenedione (**a**), testosterone (**b**), and free testosterone index (FTI) (**c**), and sex-hormone binding globulin (SHBG) (**d**) in women with polycystic ovary syndrome (PCOS), treated with metformin versus placebo in pregnancy, according to fetal sex.

Throughout pregnancy in the placebo group, women with PCOS and carrying a male or female fetus had similar A4, T, and FTI levels. Metformin significantly reduced A4 (*P* = 0.036), T (*P* = 0.023), and SHBG (*P* = 0.010) circulating levels throughout pregnancy in PCOS mothers with a male fetus, while it did not have any effect on A4, T, or SHBG levels in women with PCOS with a female fetus. Free testosterone index was not significantly altered by metformin in mothers carrying either a male or female fetus (Fig. 4A–4D).

## Discussion

The main findings of this study are that (1) pregnant women with PCOS had higher levels of A4, T, and FTI compared with healthy control women, in the first half of pregnancy, (2) A4 and T levels were relatively stable, while FTI decreased throughout pregnancy in women with PCOS, and (3) metformin had no androgen-lowering effect in the total PCOS study population; however, (4) in subgroup analyses, metformin lowered A4 levels in nonobese PCOS women, alone, as well as A4 and T levels in those carrying a male fetus.

Higher maternal androgen levels during gestation was expected in women with PCOS, as their gestational hyperandrogenicity is well-documented, likely due to their preconception androgen excess contributing to a PCOS diagnosis. Our findings are supported by a study of Sir-Petermann and colleagues (27), in which second trimester A4, T, Dehydroepiandrosteron, and free androgen index were higher in women with PCOS compared with healthy women. Caanen et al (28) reported higher T and A4 both in mid-pregnancy and at delivery. Glintborg et al (29) found higher T and FT and Piltonen et al (30) found higher T and A4, all in the third trimester in PCOS mothers compared with healthy mothers.

### Metformin effect

Metformin demonstrated androgen-lowering effects during pregnancy, as reported in a case report and in a nonrandomized study (31). In our pilot study with 40 participants, no effect of metformin on androgens was observed (32). In that study, however, a less sensitive method of ELISA analysis was used to quantify androgen levels. In a meta-analysis report, metformin had no lowering effect on free androgen index in nonpregnant women with PCOS (33). The mild T lowering effect of metformin, reported in nonpregnant PCOS women (33) was not confirmed in pregnancy.

### BMI categories

In subgroup analyses, based on BMI categories, we found that metformin significantly lowered A4 and tended to lower T levels among women with BMI ˂ 30 kg/m^2^. In a Cochrane meta-analysis, metformin demonstrated a stronger T-lowering effect on nonobese, nonpregnant women with PCOS compared with obese nonpregnant PCOS women (34, 35). This effect is most probably due to metformin inhibiting *HSD3B2* and 17,20 lyase activity of *CYP17A1*. The same mechanism may be applicable for the pregnant state. Lower plasma and tissue concentrations of metformin in obese women with PCOS may contribute to its obesity-diminished impact, due perhaps, in part, to an unvarying and not body mass index-corrected metformin dosage given to all participants.

### Fetal sex

Women with PCOS, pregnant with either a male or a female fetus, had similar A4, T, and FTI through pregnancy. This is consistent with an earlier study that reported similar levels of Dehydroepiandrosteron, T, SHBG, FTI, and T at delivery for 20 pregnant women with PCOS who gave birth to 14 girls and 6 boys (27). Surprisingly, we show that the introduction of metformin only lowered A4 and T levels in mothers carrying a male fetus, but had no effect on mothers carrying a female fetus. Whether, or to what extent, decreased A4 and T levels are reflected in fetal circulation is not known. The finding of lowered androgen levels in pregnancies with male fetuses, when metformin is introduced, should be interpreted with great caution, due to reduced sample size of the subgroup analyses and borderline *P*-values.

While contributions of the sex of fetus to differential maternal hormonal responses to metformin were unanticipated, human male fetuses can exhibit more severe placental histopathological lesions than found in females (36). Sex differences have been found in human placental perinatal responses (37), and placental epigenomic and transcriptomic profiles vary by fetal sex (38).

### Strengths and limitations

A strength of this study is the placebo-controlled randomized controlled trial design and the large number of PCOS participants. The trial was performed during routine clinical practice, and participants were representative for the population of women with a known diagnosis of PCOS. The longitudinal measurements of androgens throughout pregnancy is also a strength, including the re-analysis of samples with highly specific liquid chromatography-mass spectrometry. Adherence to study medication during pregnancy was high, and the original study was conducted in accordance with “Good Clinical Practice Principles” (24). A limitation of this study includes limited quantitative assessments of maternal steroid hormones. It would have been useful to quantify other androgens and steroid sex hormones through pregnancy. In addition, the control group lacked serum samples from the third trimester of pregnancy and gestational time points for serum sampling from controls and PCOS women were not identical. The statistics used, however, together with the presentation of data from healthy controls, provided quantitative and a visual understanding of between-group differences.

## Conclusion

The present study confirms that circulating maternal androgen levels are higher in PCOS compared with healthy pregnant women. Metformin had no effect on maternal androgens in PCOS pregnancies. In subgroup analyses, however, a modest androgen-lowering effect was observed in nonobese, pregnant women with PCOS and in those carrying a male fetus. It is not known whether the androgen-lowering effects of metformin also occur in the fetal circulation.

## Data Availability

The datasets generated during and/or analysed during the current study are not publicly available but are available from the corresponding author on reasonable request.

## Additional Information

***Disclosure Summary:*** The authors have nothing to disclose.
